# Immune landscape and a novel immunotherapy-related gene signature associated with clinical outcome in early-stage lung adenocarcinoma

**DOI:** 10.1007/s00109-020-01908-9

**Published:** 2020-04-25

**Authors:** Xuanwen Bao, Run Shi, Tianyu Zhao, Yanfang Wang

**Affiliations:** 1 Institute of Radiation Biology, Helmholtz Center Munich, German Research Center for Environmental Health, Neuherberg, Oberschleißheim, Germany; 2 Technical University Munich (TUM), Munich, Germany; 3 Department of Radiation Oncology, University Hospital, Ludwig Maximilian University of Munich, Munich, Germany; 4 Institute and Clinic for Occupational, Social and Environmental Medicine, University Hospital, Ludwig Maximilian University of Munich; Comprehensive Pneumology Center (CPC) Munich, member DZL; German Center for Lung Research, Munich, Germany; 5 Institute of Epidemiology, Helmholtz Zentrum München, German Research Center for Environmental Health, Neuherberg, Oberschleißheim, Germany; 6 Ludwig-Maximilians-Universität München (LMU), Munich, Germany

**Keywords:** Early-stage lung adenocarcinoma (LUAD), Immune landscape, Prognostic model, Overall survival

## Abstract

**Abstract:**

Patients with early-stage lung adenocarcinoma (LUAD) exhibit different overall survival (OS) rates and immunotherapy responses. Understanding the immune landscape facilitates the personalized treatment of LUAD. The immune cell populations in tumour tissues were quantified to depict the immune landscape in early-stage LUAD patients in The Cancer Genome Atlas (TCGA). Early-stage LUAD patients in three immune clusters identified by the immune landscape exhibited different survival potentials. A prognostic immune-related gene signature was built to predict the survival of early-stage LUAD patients. Several machine learning methods (support vector machine, naive Bayes, random forest, and neural network-based deep learning) were applied to train the classifiers to identify the immune clusters in early-stage LUAD based on the gene signature. The four classifiers exhibited a robust effect in identifying the immune clusters. A random forest regression model identified that TP53 was the most important gene mutation associated with the immune-related signature. Furthermore, a decision tree and a nomogram were constructed based on the immune-related gene signature and clinicopathological traits to improve risk stratification and quantify risk assessment for individual patients. Five external test cohorts were applied to validate the accuracy of the immune-related signature. Our study might contribute to the development of immunotherapy and the personalized treatment of early-stage LUAD.

**Key messages:**

- Immune landscape correlates with the clinical outcome of early-stage adenocarcinoma (LUAD).
- Machine learning methods identifies a prognostic gene signature to predict the survival and prognosis of early-stage LUAD.
- TP53 gene mutation status correlates with the immune landscape in early-stage LUAD.

**Electronic supplementary material:**

The online version of this article (10.1007/s00109-020-01908-9) contains supplementary material, which is available to authorized users.

## Introduction

Lung adenocarcinoma (LUAD) is one of the most complex and heterogeneous malignancies. Its incidence has been increasing in younger cohorts of males and females in recent years [1]. Low-dose computerized tomography (CT) screening and positron emission tomography/CT screening help the early diagnosis of LUAD. Therefore, stage I non-small cell lung cancer patients have 5-year survival rates of 83% (stage IA) and 71% (stage IB) [2]. However, the survival rate drops to 50% for stage II patients due to the minimal improvement of adjuvant chemotherapy [2]. Thus, identifying a new strategy for the treatment of early-stage LUAD is urgent. One study showed an impressive treatment effect by anti-PD-1 mAb blockade in a small number of early-stage non-small cell lung cancer patients in the neoadjuvant setting [3]. Moreover, some studies revealed tremendous benefits from the checkpoint blockade therapy in patients with a low tumour burden [4–6]. Hence, the immune checkpoint blockade therapy serves as a promising therapeutic strategy for early-stage LUAD. In addition, increasing evidence indicates the important roles of the immune microenvironment in the progression and malignancy of cancers and in affecting the immunotherapy response [4, 7, 8]. A detailed understanding of the immune microenvironment may aid in the development of immunotherapy strategies.

In this study, we identified the immune landscape of early-stage LUAD. Three clusters were obtained by unsupervised clustering of the abundance of immune cell populations in early-stage LUAD tissues. Key immune-related genes were identified by the differentially expressed gene (DEG) analysis among the three clusters. An immune-related gene signature was built and assessed by several computational biology and machine learning methods. Through detailed bioinformatics analyses of RNA-seq data and clinical data, we describe the association of the immune gene signature and prognosis of early-stage LUAD patients.

## Methods

### Dataset preparation and data processing

The Cancer Genome Atlas (TCGA) RNA-seq datasets and clinical data from LUAD patients were downloaded from the UCSC Xena browser (https://xenabrowser.net/). GSE42127, GSE37745, GSE50081, GSE29013, and GSE72094 were downloaded from the GEO database (http://www.ncbi.nlm.nih.gov/geo/). Early-stage LUAD patients with TNM stages I–II were filtered by the criteria. CCLE cell line transcriptome data were downloaded from the CCLE database (https://portals.broadinstitute.org/ccle).

### Implementation of single-sample gene set enrichment analysis and gene ontology analysis

Single-sample gene set enrichment analysis (ssGSEA) was performed to derive the enrichment scores of each immune-related cell population by the R package GSVA [9]. The computational approach used in our study included immune cell types that are involved in innate immunity and adaptive immunity [10]. The hallmark gene sets were obtained from the Molecular Signatures Database (MSigDB). Gene ontology (GO) analysis was performed by using the clusterProfiler package [7].

### Differently expressed gene analysis

DEG analysis was performed by using the limma package [11]. An empirical Bayesian approach was applied to estimate the gene expression changes using moderated *t* tests. The adjusted *p* value for multiple testing was calculated using the Benjamini-Hochberg correction. The DEGs were defined as genes with an adjusted *p* value less than 0.01.

### LASSO Cox regression

LASSO (least absolute shrinkage and selection operator) is an important regularization in many regression analysis methods. Here, we applied LASSO Cox regression. L1-norm was applied to penalize the weight of the model parameters. Unimportant parameters shrunk to zero, and the remaining genes were used to build a gene signature. An immune-related signature-based risk score formula was established by including individual normalized gene expression values weighted by their LASSO Cox coefficients:

$$\sum \limits_i\mathrm{Coefficient}\left({\mathrm{mRNA}}_i\right)\times \mathrm{Expression}\left({\mathrm{mRNA}}_i\right)$$

### Classifier construction

The TCGA cohort was randomly divided into training sets and testing sets. The training sets were applied to train the three classifiers. The object of support vector machine (SVM) was to find a hyperplane in an N-dimensional space that distinctly classifies the data points. Hyperplanes are decision boundaries that help classify the data points. Support vectors are points that are closer to the hyperplane and influence the position and orientation of the hyperplane. Hinge loss is applied to optimize the maximum margin. Random forest is a supervised learning algorithm. It can be used both for classification and regression. In our analysis, we used a random forest classification method for classifier construction and a random forest regression method for the analysis of the association between gene mutations and the immune-related gene signature. Naive Bayes is a probabilistic classifier that is based on the Bayes theorem. The neural network was performed with an activation function (Python script for the construction of the neural network is in Doc. S1). We built the four classifiers to predict the immune clusters in the analysis.

### Random forest algorithm for feature importance ranking

A random forest algorithm was applied to find the most critical mutations associated with the immune signature-based risk score [12]. Briefly, the gene mutation dataset and immune cell signature-based risk score were applied to find the most important gene mutations associated with the immune signature-based risk score. The ranger package was used to find the best hyperparameter in the regression process and build the model [13].

### Decision tree and nomogram construction

A diagnostic decision tree was constructed with the rpart package [14]. The trimming parameter was set as default. A nomogram was constructed with the rms package [15]. Multiple Cox regression was performed to construct the nomogram.

### Immunotherapy response prediction

The TIDE website (http://tide.dfci.harvard.edu/) was used to predict the response to immunotherapy.

## Results

### Immune landscape of early-stage LUAD

**Fig. 1:**
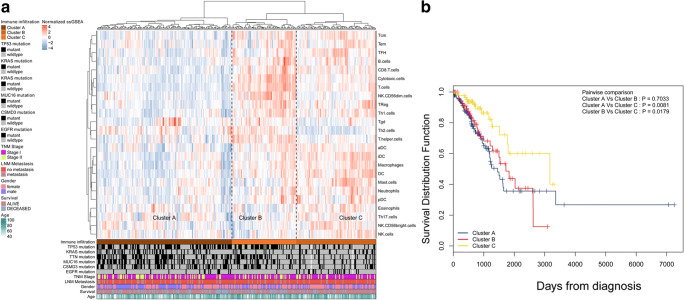
Immune landscape of early-stage LUAD and TME characteristics. **a** Unsupervised clustering of early-stage LUAD tissues using ssGSEA scores from immune cell types. The mutation status of TP53, KRAS, TTN, MUC16, CSMD3, and EGFR; survival; sex; age; lymph node metastasis; and TNM stage are shown as patient annotations in the lower panel. Hierarchical clustering was performed with Euclidean distance and Ward linkage. Three distinct immune infiltration clusters, termed cluster A, cluster B, and cluster C, were defined. **b** Kaplan-Meier curves for the OS of early-stage LUAD patients showed that the cluster C group had a favourable outcome compared with the other groups. TME, tumour microenvironment; TCGA, The Cancer Genome Atlas; OS, overall survival

**Fig. 2:**
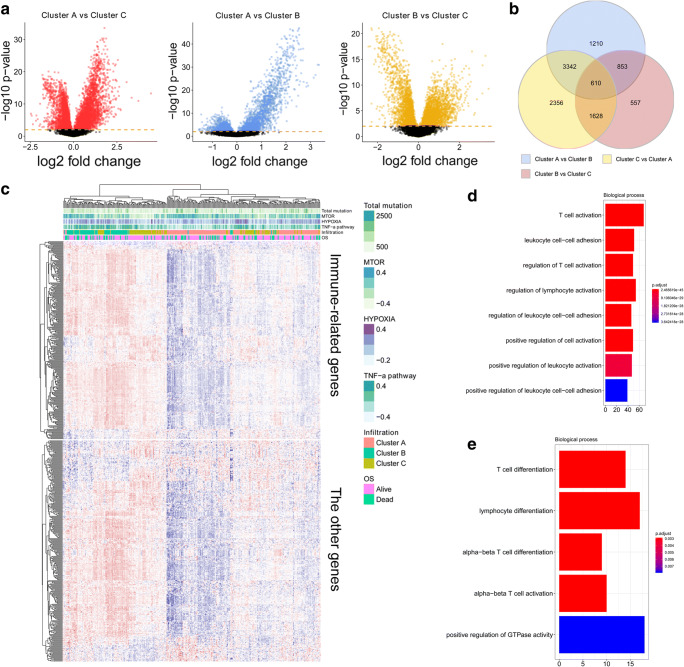
DEG analysis identified key immune-related genes. **a** The volcano plot showing the DEGs among different clusters. **b** The Venn diagram showing the key immune-related genes by the overlapping of DEGs between cluster A and cluster B, cluster B and cluster C, and cluster A and cluster C. **c** The heatmap showing the expression level of the immune-related genes and other genes in the TCGA cohort. **d** GO analysis based on the immune-related genes. **e** GO analysis based on other genes

The early-stage LUAD cohort was obtained from the TCGA LUAD cohort (Supplementary Table 1). The immune landscape of early-stage LUAD was depicted with several immune cell populations. The abundance of 24 immune cell populations was estimated by the ssGSEA algorithm. The early-stage LUAD tumour tissues were clustered into 3 clusters (clusters A, B, and C) by using the hierarchical clustering method (Fig. 1a). The association of overall survival (OS) with different clusters of early-stage LUAD was analyzed by a pairwise log-rank test (Fig. 1b). The results indicated that cluster C had a favourable survival probability compared with cluster A and cluster B. DEG analysis was performed for the three clusters. The volcano plot in Fig. 2a shows the DEGs among each of the clusters. The Venn diagram in Fig. 2b shows that 610 genes overlapped among all three clusters. Among the 610 genes, 271 genes were immune-related genes (Doc. S2, filtered by immune-related gene list from https://www.innatedb.com/redirect.do?go=resourcesGeneLists). The expression levels of the 610 genes are exhibited in the heatmap in Fig. 2c. Unsupervised clustering showed similar clustering results compared with the clustering by immune cell populations, which revealed the potential association of the 610 genes with the immune cell population in early-stage LUAD. GO analysis was performed based on the 271 immune-related key regulators in early-stage LUAD and 271 other genes (Fig. 2d and e). The results indicated that T cell activation and T cell differentiation were the most significant terms, which further confirmed the association of the 610 overlapping genes with immune regulation in early-stage LUAD.

### Prognostic model construction

**Fig. 3:**
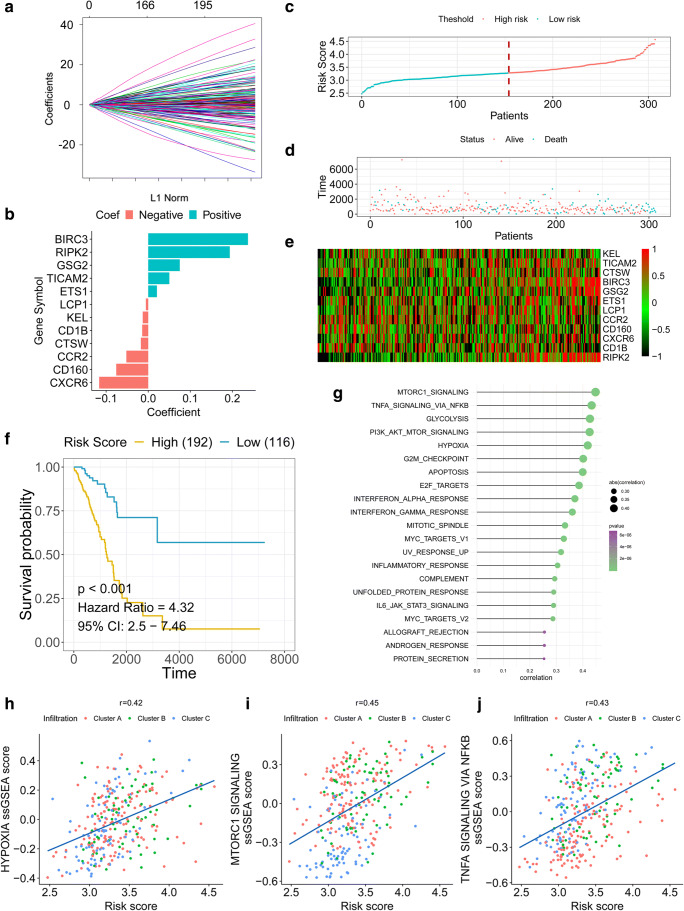
Construction of a prognostic immune-related gene signature. **a** LASSO Cox regression with L1 regularization. **b** Distribution of LASSO coefficients of the hypoxia-related gene signature. **c** Risk score distribution for each patient. **d** Survival overview. **e** Heatmap showing the expression profiles of the signature in low- and high-risk groups. **f** Patients in the high-risk group exhibited worse overall survival than those in the low-risk group. **g** ssGSEA revealed the most significant hallmarks correlated with the immune-related signature. **h** The correlation between the hypoxia ssGSEA score and immune-related signature-based risk score. **i** The correlation between mTORC1 signalling ssGSEA score and immune-related signature-based risk score. **j** The correlation between TNF-α signalling ssGSEA score and immune-related signature-based risk score

**Fig. 4:**
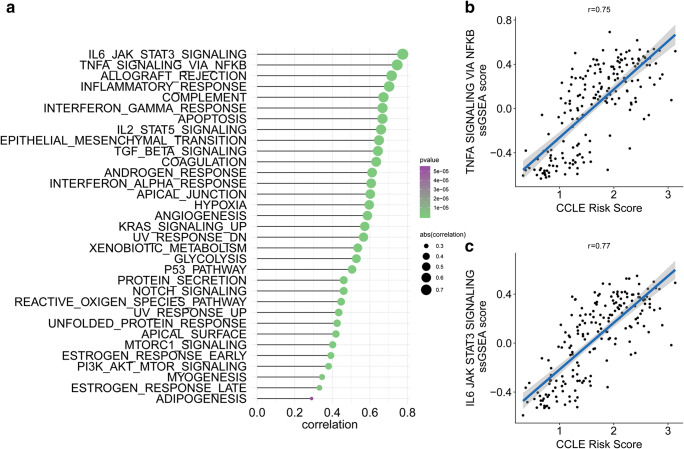
The correlation between the ssGSEA score and immune-related signature-based risk score in CCLE lung cancer cell lines. **a** ssGSEA revealed the most significant hallmarks correlated with the immune-related signature in CCLE lung cancer cell lines. **b** The correlation between the TNF-α signalling ssGSEA score and immune-related signature-based risk score. **c** The correlation between the IL-6/JAK/STAT3 signalling ssGSEA score and immune-related signature-based risk score

The 271 immune-related regulators were used to build a prognostic model to predict the OS of early-stage LUAD patients. LASSO Cox regression penalized the unimportant features in the regularization process, and 12 features (BIRC3, RIPK2, GSG2, TICAM2, ETS1, LCP1, KEL, CD1B, CTSW, CCR2, CD160, and CXCR6) were finally selected for the final model construction (Fig. 3a–b). The risk score for each patient was calculated based on the expression level of the 12 genes with the coefficient in the model. The risk score formula was established as follows: $\sum \limits_i\mathrm{Coefficient}\left({\mathrm{mRNA}}_i\right)\times \mathrm{Expression}\left({\mathrm{mRNA}}_i\right)$. The early-stage LUAD patients were separated into a high-risk group and a low-risk group by the median cut-off value (Fig. 3c–d). The heatmap in Fig. 3e shows the expression level of the 12 genes. The KM plot revealed that early-stage LUAD patients with a low-risk score had a favourable survival outcome compared with patients with a high-risk score (HR = 4.32, *p* < 0.001) (Fig. 3f). ssGSEA was performed for each patient in the early-stage LUAD cohort (Fig. 3g). The results revealed that MTOR1 signalling, TNF-α signalling via NF-KB, hypoxia, and several other pathways were highly related to the immune-related gene signature-based risk score by using the Spearman’s test. Hypoxia, which plays crucial roles in drug resistance in LUAD, had a coefficient of 0.42 with the immune-related gene signature-based risk score (Fig. 3h). MTOR1 signalling, which contributes to the malignancy and tumour progression of LUAD, had a coefficient of 0.45 with the immune-related gene signature-based risk score (Fig. 3i). TNF-α signalling via NF-KB, which is involved in the metastasis and angiogenesis of LUAD, had a coefficient of 0.43 with the immune gene signature (Fig. 3j). To further identify the potential role of the immune-related signature-based risk score in LUAD, we also performed ssGSEA in CCLE lung cancer cell lines (Fig. 4a). The Spearman’s test revealed a high correlation between the immune-related gene signature-based risk score and TNF-α signalling via NF-kB or immune-related pathways, which further confirmed the findings in TCGA tumour tissues (Fig. 4b–c).

### Construction of four classifiers for identifying the immune status

**Fig. 5:**
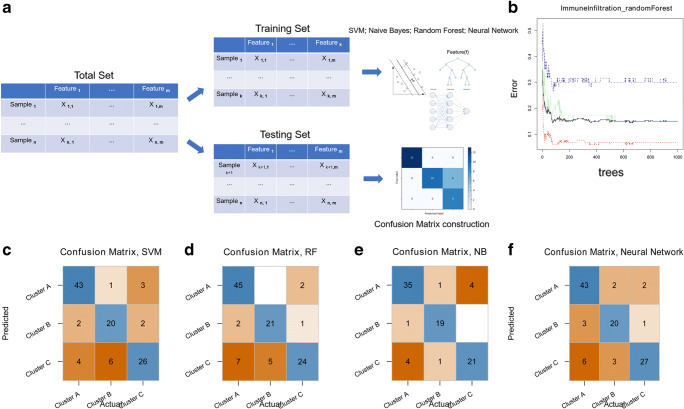
Classifier construction based on key immune-related genes. **a** Schematic diagram for training and testing the classifiers. **b** The relationship between tree building and the error rate in the random forest classifier. **c** The confusion matrix for the random forest classifier. **d** The confusion matrix for the naive Bayes classifier. **e** The confusion matrix for the support vector machine classifier. **f** The confusion matrix for the neural network classifier

The early-stage LUAD cohort was randomly divided into training and testing data sets. Four classifiers, which included naive Bayes, random forest, support vector machine, and neural network-based deep learning, were trained based on the transcriptome data of the immune-related gene signature (Fig. 5a). The confusion matrix depicted the prediction accuracy of the three classifiers in the testing data set. Figure 5b illustrates the accuracy of the random forest classifier. The average prediction accuracy of the four classifiers was above 0.8 in the testing set, indicating robust efficiency in identifying different immune clusters in early-stage LUAD by the four classifiers (Fig. 5c–f). The results above confirmed the importance of the immune-related gene signature in the immune microenvironment of early-stage LUAD.

### Correlation between the immune-related signature and somatic gene mutations

**Fig. 6:**
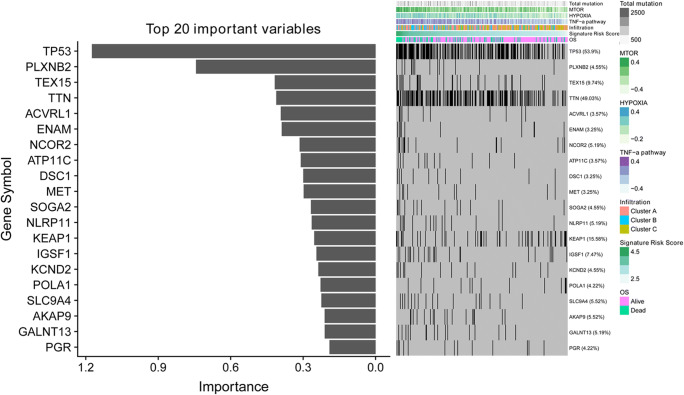
The association of the immune signature with early-stage LUAD gene mutations. **a** The correlation between the immune signature and early-stage LUAD gene mutations. **b** Distribution of gene mutations correlated with the immune signature

The Spearman test revealed that the correlation coefficient was 0.15 between the immune-related gene signature-based risk score and total mutations (Fig. 6a). A random forest regression algorithm was used to identify the correlation between somatic gene mutations and the immune gene signature. A feature importance method was applied to reveal key somatic gene mutations that are highly associated with the immune-related gene signature. Among the 20 most important features, TP53 is the gene mutation with the greatest feature importance (Fig. 6b).

### Subgroup survival analysis

**Fig. 7:**
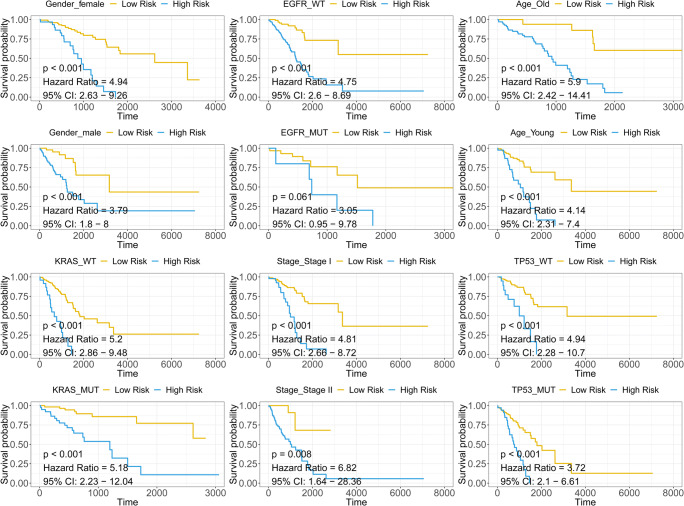
The subgroup survival analysis. *p* < 0.05 is considered as significant. HR, CI, and *p* value for univariate Cox analysis is shown in each survival plot

The immune-related signature serves as a promising marker to predict overall survival in different subgroups, including female (HR = 4.94, CI 2.63–9.26, *p* < 0.001), male (HR = 3.79, CI 1.8–8, *p* < 0.001), KRAS-wild type (KRAS-WT) (HR = 5.2, CI 2.23–12.04, *p* < 0.001) and KRAS-mutated (KRAS-Mut) (HR = 5.18, CI 2.23–12.04, *p* < 0.001), EGFR-wild type (EGFR-WT) (HR = 4.75, CI 2.6–8.69, *p* < 0.001), EGFR-mutated (EGFR-Mut) (HR = 3.05, CI 0.95–9.78, *p* = 0.061), stage I (HR = 4.81, CI 2.66–8.72, *p* < 0.001), stage II (HR = 6.82, CI 1.64–28.36, *p* = 0.008), old (> 60) (HR = 5.9, CI 2.42–14.41, *p* < 0.001), young (< 60) (HR = 4.14, CI 2.31–7.4, *p* < 0.001), TP53-wild type (TP53-WT) (HR = 5.9, CI 2.28–10.7, *p* < 0.001), and TP53-mutated (TP53-Mut) (HR = 3.72, CI 2.1–6.61, *p* < 0.001) patients (Fig. 7).

### Risk stratification of early-stage LUAD patients by decision tree and nomogram

**Fig. 8:**
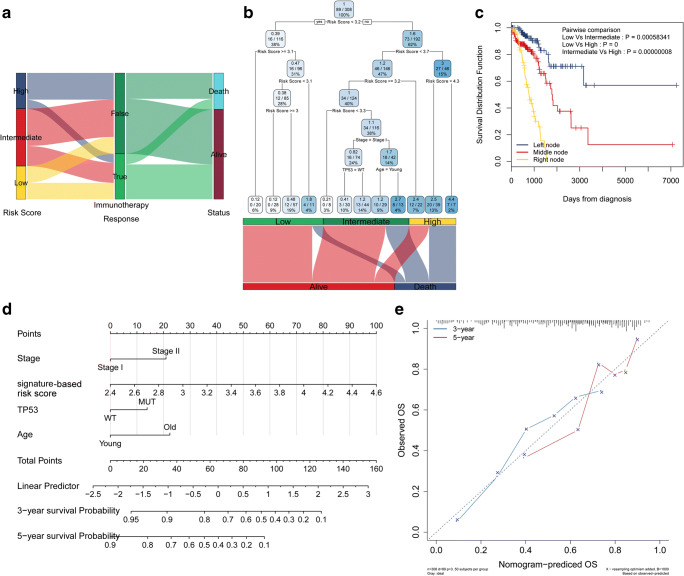
Combination with clinicopathological traits to improve risk stratification and survival prediction. **a** The association between the immune-related signature-based risk score and immunotherapy response. **b** A decision tree was constructed to improve risk stratification. **c** OS differed markedly among the three risk subgroups from the decision tree. **d** A nomogram was constructed to quantify risk assessment for individual patients. **e** Calibration analysis indicated a high accuracy for survival prediction

The transcriptome data from patients who did not receive immunotherapy were used to predict the response to immunotherapy by TIDE. Sankey plots showed that patients with high immune-related signature-based risk scores had poor responses to immunotherapy and unfavourable survival outcomes (Fig. 8a). Univariate and multivariate Cox analyses were performed with immune signature-based risk score and other clinicopathological features (TNM stage, TP53 mutation status, EGFR mutation status, KRAS mutation status, gender, age). Results revealed that the risk score had greater HR than TNM stage, age, and TP53 mutation status in both univariate and multivariate Cox analysis, which indicated that the immune signature-based risk score may be a promising predictor (Supplementary Table 2). A decision tree was constructed to improve risk stratification for overall survival in early-stage LUAD patients (Fig. 8b). TNM stage, TP53 mutation status, age, and immune-related gene signature-based risk score were applied to build the decision tree, with three different risk subgroups identified. Three clusters (low-risk, intermediate-risk, and high-risk groups) were identified as the outcome of the decision tree. As shown in the Kaplan-Meier curve in Fig. 8c, overall survival differed markedly among the three risk subgroups.

With the goal of quantifying the risk assessment and survival probability for individual early-stage LUAD patients, a nomogram was generated with the immune-related gene signature-based risk score together with other clinicopathological traits (TNM stage, age, and TP53 mutation status) (Fig. 8d). The prediction line (red line 3-year survival probability prediction, and blue line 5-year survival probability prediction) of the nomogram was close to the ideal performance (45-degree dotted line) in the calibration analysis (Fig. 8e), suggesting a high level of accuracy of the nomogram (Fig. 8d).

### Validation of the immune-related gene signature in external cohorts

**Fig. 9:**
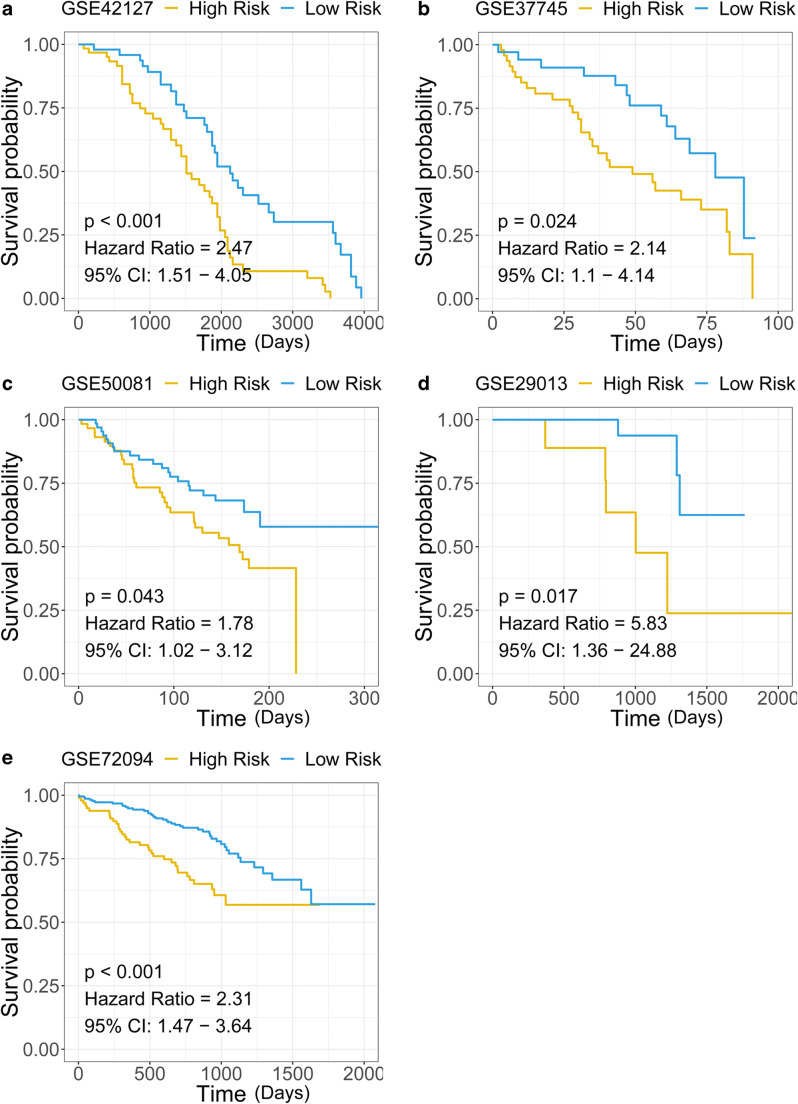
The gene signature serves as a valuable marker for poor survival in several external cohorts. **a**–**e** Patients with higher risk scores exhibited worse overall survival among different external cohorts

Five GEO datasets (GSE42127, GSE37745, GSE50081, GSE29013, and GSE72094) were used to validate the prognostic effect of the immune-related gene signature. Kaplan-Meier analysis and Cox regression were applied in each cohort, showing the risk stratification capacity of the immune-related gene signature for survival prediction (Fig. 9a–e).

## Discussion

Increasing evidence shows that the immune microenvironment is involved in the progression and malignancy of LUAD [16–19]. Immunotherapy serves as a promising therapeutic strategy for early-stage cancer patients with a low tumour burden. The objective of this study was to depict the immune landscape of early-stage LUAD and establish a prognostic and immunotherapy-relevant gene signature. With the help of several bioinformatic and machine learning methods, we identified and assessed the immune landscape of early-stage LUAD. Key immune-related genes were obtained by using the DEG analysis. A prognostic and immunotherapeutic gene signature was identified with the key immune-related genes. A random forest regression algorithm was applied to identify the key gene mutations associated with the immune-related gene signature. The decision tree and nomogram help improve the predictive power and accuracy of the model.

Early-stage LUAD tissues were clustered into three clusters. The log-rank test revealed the difference in OS of early-stage LUAD patients in the three clusters. The above results indicated the influence of the tumour immune microenvironment on the prognosis of early-stage LUAD patients. There were 610 genes obtained from the DEG analysis among the three clusters. Among the 610 genes, 271 genes were immune-related genes, which constituted a large proportion of the total of 610 genes. We performed the GO analysis for the immune-related genes and other genes. Strikingly, both results showed a relationship with immune-related enrichments. One study revealed that LUAD tumour tissues had a strongly reduced CD8+ T effector/Treg ratio compared with normal tissues. The altered T cell ratio resulted from a significant reduction in CD8+ T cells expressing granzyme B and IFNγ and from a significant expansion of CD39^hi^CD38^hi^PD-1^hi^CTLA4^hi^Foxp3^hi^ Tregs at the tumour site [18]. In our analysis, we highlighted the importance of the 610 genes in modulating T cell activation and entry into the tumour lesions.

The BIRC3 gene had the largest coefficient in the immune-related gene signature. The primary role of BIRC3 is inhibiting apoptosis by binding to the tumour necrosis factor receptor-associated factors TRAF1 and TRAF2. In recent studies, BIRC3 was identified as a gene involved in chemoresistance in breast cancer and gliomas [20, 21]. The TNF-α pathway controls the expression of BIRC3 and helps protect breast cancer cells against apoptosis [22]. In our analysis, BIRC3 is one of the most important features of the immune-related gene signature. ssGSEA showed a high correlation between the immune-related gene signature-based risk score and the TNF-α pathway. Overall, we concluded that BIRC3 is an important key regulator that cross-links the TNF-α pathway and immune response in early-stage LUAD. Considering the heterogeneity in LUAD tumour tissues, we also performed ssGSEA in lung cancer cell lines. Strikingly, Spearman’s test revealed a coefficient of 0.77 between the immune-related gene signature-based risk score and the TNF-α pathway. TNF-α pathway-mediated inflammatory responses play decisive roles in tumour development, including initiation, promotion, invasion, and metastasis. Inflammatory activities in the tumour microenvironment also affect immune surveillance and responses to therapy [23]. One study revealed that the activation of NF-κB in immune cells induces the production of cytokines that activate NF-κB in cancer cells to induce chemokines that attract more inflammatory cells into the tumour [24]. This protumorigenic feedback loop contributed to tumour progression. In early-stage LUAD, we found that TNF-α-mediated inflammation was associated with immune activity based on the high correlation between the TNF-α pathway and the immune-related signature. Disrupting the crosstalk between the TNF-α pathway and the immune response may be a potential target in the treatment of early-stage LUAD. The immune response is conventionally considered an anti-tumour mechanism. Interestingly, in our analysis of CCLE lung cancer cell lines, the immune-related gene signature-based risk score showed a high correlation with the IL-6/JAK/Stat3 pathway, which is consistent with the notion that antitumorigenic and protumorigenic immune and inflammatory mechanisms coexist in developing tumours, but if the tumour is not rejected, the protumorigenic effect dominates [24]. In the tumour microenvironment, IFN-γ, which is produced by cytotoxic CD8+ and CD4+ Th1 T cells, is considered the major anti-tumour immune effectors, whereas the cytokines IL-6, TNF, IL-1β, and IL-23, which are produced by tumour-associated macrophages or myeloid-derived suppressive cells, are generally recognized as dominant tumour-promoting factors [25]. In our analysis, the high correlation between the immune-related gene signature-based risk score and the IL-6/JAK/Stat3 pathway highlighted the tumour-promoting effect of the IL-6/JAK/Stat3 pathway in early-stage LUAD [4, 26].

To further illustrate the importance of the immune-related gene signature in early-stage LUAD, we trained four classifiers based on the transcriptome of the immune-related gene signature. SVM, random forest, naive Bayes, and neural network-based deep learning all showed high accuracy in identifying the immune clusters. Taken together, the results above revealed the strong connection between the immune-related gene signature and the immune cell populations in early-stage LUAD tissues.

TP53 is the most important feature associated with the immune-related gene signature, which was identified by using the random forest regression algorithm. TP53 mutation status has been found to be associated with immune cell infiltration in many cancers [27–29]. Patients with TP53 mutations tended to have a larger immune-related gene signature-based risk score than patients with TP53 wild type. Nonetheless, the immune-related gene signature can stratify risk in both TP53 mutation and wild type conditions. The relationship between TP53 and immune response may be a promising target in early-stage LUAD patients.

In our previous study, we found that lymph node metastasis-related biomarkers, DNA epigenetic regulation, and other clinicopathological and molecular mechanisms could affect the survival of LUAD patients [30, 31]. In this study, we highlighted the importance of the immune landscape in early-stage LUAD. Through the combination of immune-related gene signatures and other clinicopathological features, we built a decision tree and a nomogram to stratify high-risk early-stage LUAD patients. During the trimming step of the decision tree, the decision tree exhibited a strong relationship between the immune-related signature-based risk score and the OS of early-stage LUAD patients. Moreover, the immune-related signature also served as a prognostic factor when constructing the nomogram. We checked the relationship between the immunotherapy response and the immune-related gene signature. Interestingly, patients who had larger immune-related gene signature-based risk scores usually had poor immunotherapy responses, which further validates the prognostic value of the immune-related gene signature.

In this study, we depicted the immune landscape and established a novel immune-related gene signature to discriminate high-risk patients with early-stage LUAD. Through detailed bioinformatics analyses of RNA-seq data and clinical data, we confirmed that the immune gene signature is a powerful predictor. Integrated with clinicopathological traits, we built a decision tree to optimize risk stratification for OS and a nomogram to quantify risk assessment for individual patients. Our model could be a useful tool for personalized management of early-stage LUAD patients.

## Electronic supplementary material

ESM 1 (DOCX 17 kb)

## Data Availability

The datasets supporting the conclusions of this article are available in the Xena browser (https://xenabrowser.net/) repository.
