# Supplementary material for: Immune landscape and a novel immunotherapy-related gene signature associated with clinical outcome in early-stage lung adenocarcinoma
Source: J Mol Med (Berl). 2020 Apr 25;98(6):805–18. doi: 10.1007/s00109-020-01908-9 (PMC7297823; doi:10.1007/s00109-020-01908-9)
Supplement: Supplementary file 1 — (DOCX 17 kb) [file 109_2020_1908_MOESM1_ESM.docx]

**Supplementary Table 1**:

| Patient information | |
| --- | --- |
| Variable | Number |
| Gender (female/male) | 166/142 |
| TNM Stage (stage I/stage II) | 212/96 |
| Lymph node metastasis (positive/negative) | 61/247 |
| Age (>60/<=60/missing) | 221/78/9 |
| TP53 Status (WT/MUT) | 210/98 |
| Smoking (no/yes/missing) | 15/93/200 |

**Supplementary Table 2**:

| Survival analysis | | |  | |  |  | |  | |  | |  |
| --- | --- | --- | --- | --- | --- | --- | --- | --- | --- | --- | --- | --- |
|  | Univariate Cox analysis | | | | | | Mulvariate Cox analysis | | | | |  |
| Variable | HR | 95% CI | | P-value | | | HR | | 95% CI | | P-value | |
| Gender  (female vs male) | 1.04 | 0.69-1.58 | | 0.85 | | |  | |  | |  | |
| KRAS Status  (WT vs MUT) | 1.01 | 0.62-1.56 | | 0.95 | | |  | |  | |  | |
| EGFR Status  (WT vs MUT) | 1.35 | 0.73-2.48 | | 0.33 | | |  | |  | |  | |
| Age  (Old vs Young) | 1.70 | 1.10-2.58 | | 0.016* | | | 2.10 | | 1.34-3.30 | | 0.001* | |
| TP53 Status  (WT vs MUT) | 2.07 | 1.33-3.20 | | 0.001* | | | 1.58 | | 0.97-2.59 | | 0.07 | |
| TNM Stage  (Stage II vs Stage I) | 2.34 | 1.53-3.57 | | <0.0001* | | | 1.80 | | 1.28-3.13 | | 0.002* | |
| Immune signature-  based risk score | 6.35 | 3.82-10.55 | | <0.0001* | | | 4.53 | | 2.62-7.81 | | <0.0001* | |

**Doc. S1 Neural network-based deep learning framework.**

**Doc. S2** **Differently expressed immune-related genes.**
